# Task constraints and minimization of muscle effort result in a small number of muscle synergies during gait

**DOI:** 10.3389/fncom.2014.00115

**Published:** 2014-09-18

**Authors:** Friedl De Groote, Ilse Jonkers, Jacques Duysens

**Affiliations:** ^1^Department of Mechanical Engineering, Katholieke Universiteit LeuvenLeuven, Belgium; ^2^Department of Kinesiology, Katholieke Universiteit LeuvenLeuven, Belgium; ^3^Department of Research, Sint MaartenskliniekNijmegen, Netherlands

**Keywords:** simulation, musculoskeletal model, non-negative matrix factorization, muscle synergies, modularity, walking, effort minimization

## Abstract

Finding muscle activity generating a given motion is a redundant problem, since there are many more muscles than degrees of freedom. The control strategies determining muscle recruitment from a redundant set are still poorly understood. One theory of motor control suggests that motion is produced through activating a small number of muscle synergies, i.e., muscle groups that are activated in a fixed ratio by a single input signal. Because of the reduced number of input signals, synergy-based control is low dimensional. But a major criticism on the theory of synergy-based control of muscles is that muscle synergies might reflect task constraints rather than a neural control strategy. Another theory of motor control suggests that muscles are recruited by optimizing performance. Optimization of performance has been widely used to calculate muscle recruitment underlying a given motion while assuming independent recruitment of muscles. If synergies indeed determine muscle recruitment underlying a given motion, optimization approaches that do not model synergy-based control could result in muscle activations that do not show the synergistic muscle action observed through electromyography (EMG). If, however, synergistic muscle action results from performance optimization and task constraints (joint kinematics and external forces), such optimization approaches are expected to result in low-dimensional synergistic muscle activations that are similar to EMG-based synergies. We calculated muscle recruitment underlying experimentally measured gait patterns by optimizing performance assuming independent recruitment of muscles. We found that the muscle activations calculated without any reference to synergies can be accurately explained by on average four synergies. These synergies are similar to EMG-based synergies. We therefore conclude that task constraints and performance optimization explain synergistic muscle recruitment from a redundant set of muscles.

## 1. Introduction

Walking is generated through the coordinated action of many muscles. The number of muscles largely exceeds the number of degrees of freedom and hence the musculoskeletal system is highly redundant. Because of this redundancy, a given walking pattern, characterized by joint kinematics and reaction forces between the ground and the feet, can be generated by infinitely many possible muscle recruitment strategies. A common objective of many researchers is to understand the control strategies determining muscle recruitment from a redundant set of muscles.

One theory of motor control suggests that the central nervous system produces movement through activating a small number of muscle synergies (Lee, 1984). Muscle synergies are muscle groups that are activated in a fixed ratio by a single input signal. From a control perspective, muscle synergies provide significant dimensionality reduction by limiting the achievable muscle activity patterns. Hence, controlling muscle synergies is thought to be simpler than controlling individual muscles. The hypothesis of synergy-based or modular control has mainly been studied through analyzing electromyographic (EMG) activity of a subset of muscles measured during a variety of tasks. Subsequently, computational methods such as non-negative matrix factorization (NNMF), factor analysis, or independent component analysis are used to identify a set of synergies (for a comparison of methods see Ivanenko et al., 2005; Tresch et al., 2006). For gait, three to six synergies have been shown to describe muscle activity (Patla, 1985; Davis and Vaughan, 1993; Olree and Vaughan, 1995; Ivanenko et al., 2004; Clark et al., 2010; Zelik et al., 2014). In addition, these synergies have been shown to be robust across individuals and walking conditions (e.g., walking speed and body weight support) (Ivanenko et al., 2004, 2005; Clark et al., 2010). More recently, d'Avella and Pai (2010) proposed a new approach to assess the hypothesis of synergy-based modular control based on the adaptation rate to perturbations that are either compatible or incompatible with a modular control architecture. Berger et al. (2013) found that adaptations to compatible perturbations were faster than adaptation to incompatible perturbations in reaching tasks in human subjects and conclude that this observation supports the hypothesis of modular control.

The theory of synergistic muscle control is, however, under debate. A major criticism on the theory of modular control is that the synergies or modules might reflect task constraints rather than a neural control strategy (Kutch et al., 2008; Tresch and Jarc, 2009; Valero-Cuevas et al., 2009). In other words, according to this criticism synergies reflect the fact that there are only a few ways a task can be successfully performed, once all the task constraints are fully accounted for. Kutch and Valero-Cuevas (2012) demonstrated that also non-neural constraints can produce a dimensionality reduction. As a part of their study, they used a model of the lower limb actuated by 14 muscles to investigate the dimensionality of an isometric force task at the foot. They found that the set of muscle recruitment patterns associated with isometric forces at the foot in all directions is of dimension seven, which is a considerable dimensionality reduction with respect to 14 independent muscles. However, the dimensionality reduction observed during gait based on the EMG of a similar number of muscles is larger.

Another theory of motor control suggests that muscles are recruited from a redundant set by optimizing a performance criterion. Optimization of a performance criterion has been widely used to calculate the muscle recruitment underlying a measured motion (Crowninshield and Brand, 1981; Anderson and Pandy, 2001). Such optimization approaches are based on a musculoskeletal model with independently controlled muscles and use the task constraints (joint kinematics and reaction forces between the ground and the feet) as an input. If synergies indeed determine muscle recruitment underlying a given motion, optimization approaches that do not model synergy-based control could result in muscle activations that do not show the synergistic muscle action observed through EMG. If, however, synergistic muscle action results from performance optimization and task constraints (joint kinematics and external forces), such optimization approaches are expected to result in low-dimensional synergistic muscle activations that are similar to EMG-based synergies. Hence, confronting synergies that are obtained from calculated activations with synergies that are obtained from EMG allows further investigation of the hypothesis of synergistic muscle control. In contrast to methods that are based on the decomposition of measured EMG only, the use of a musculoskeletal model to calculate muscle activity allows investigating whether task constraints and performance optimization can explain synergistic muscle recruitment underlying a given motion.

Although synergistic muscle action during gait has not yet been studied by decomposing model-based muscle activities, synergies observed through EMG have been used as inputs to model-based analysis of gait (Neptune et al., 2009; Allen and Neptune, 2012; Allen et al., 2013; Walter et al., 2014). Neptune et al. (2009) have demonstrated that walking can result from low-dimensional, synergistic muscle action. Kargo et al. (2010) confirmed this finding for wipe trajectories in the spinal frog. Walter et al. (2014) found that the accuracy of optimization methods to calculate muscle recruitment underlying gait benefits from using muscle synergies derived from experimental EMG data. This finding has also been reported for balancing tasks in the cat (McKay and Ting, 2012) and for isometric force generation at the hand (Borzelli et al., 2013). These studies demonstrate that performance optimization and synergistic muscle action can occur simultaneously but they do not allow determining whether synergistic muscle action follows from performance optimization and task constraints. In other words, the improvement in accuracy might result from the use of additional input data (EMG) rather than from imposing a control structure (synergistic muscle control).

The aim of this study was to investigate whether the synergistic muscle action observed during gait can be explained by the combination of task constraints and the minimization of muscle effort. We therefore used a musculoskeletal model to calculate the muscle recruitment underlying experimentally measured gait patterns by minimizing muscle effort while assuming independent recruitment of individual muscles. Consequently, we decomposed the calculated muscle activitions using NNMF. We found that the muscle activations calculated without any reference to synergies can be accurately reconstructed by the combination of a small number of muscle synergies that are similar to EMG-based synergies.

## 2. Materials and methods

### 2.1. Experimental apparatus and data acquisition

**Figure 1:**
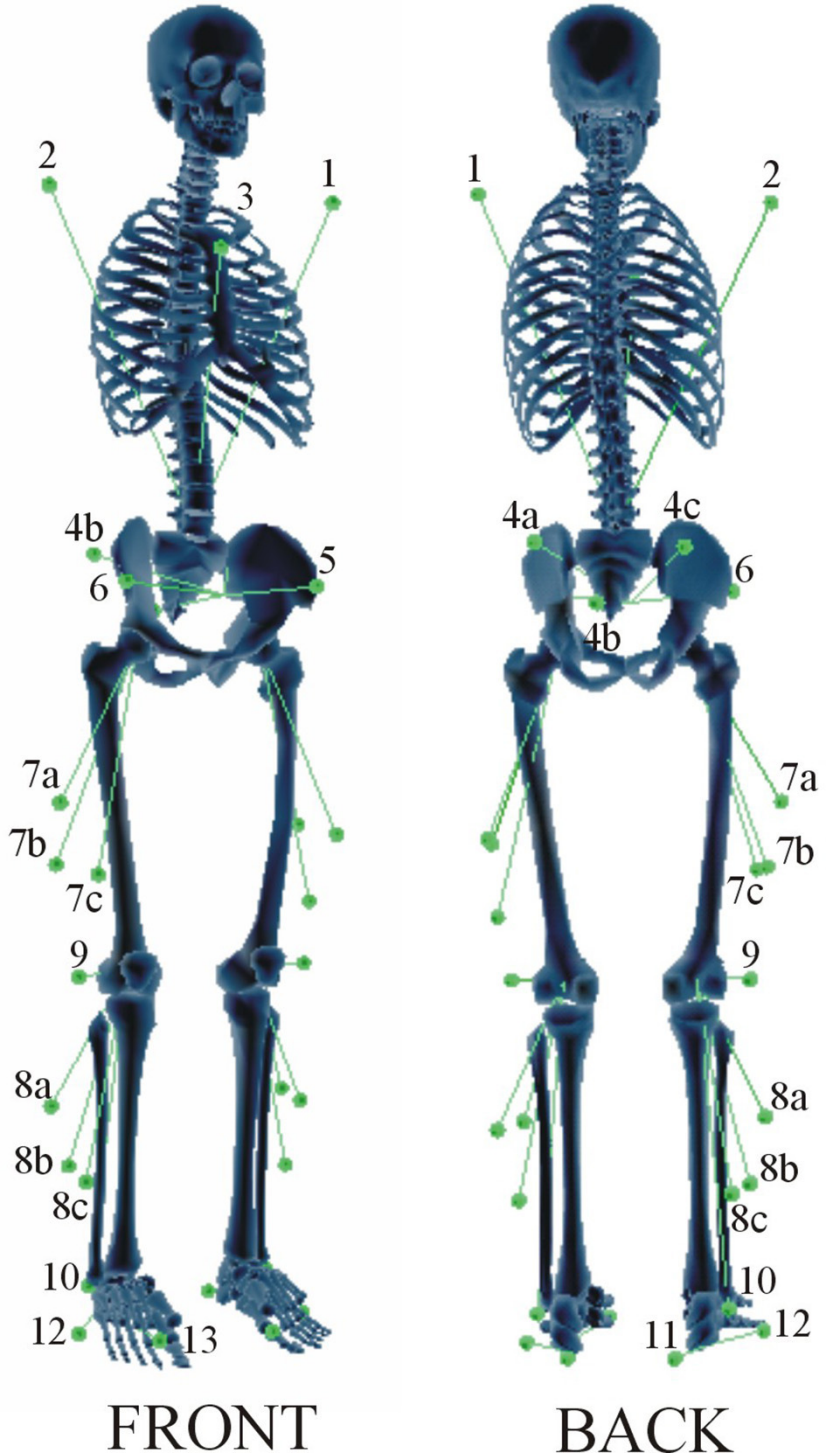
**Marker placement protocol**. A modified Cleveland marker placement protocol (Sutherland, 2002) was used for the data collection. The marker set consisted of 30 markers, including five clusters of three markers. Three anatomical markers defined the trunk: a marker on the lateral aspects of the left (1) and right (2) shoulder and a marker on the sternum (3). The pelvis segment is defined by a cluster of three technical markers on the sacrum (4a, 4b, 4c) and two anatomical markers on the left (5) and right (6) Anterior Superior Iliac Spine (ASIS). The thigh segment is defined by a cluster of three technical markers (7a, 7b, 7c). The shank segment is defined by a cluster of three technical markers (8a, 8b, 8c), an anatomical marker on the lateral epicondyle (9), and an anatomical marker on the lateral malleolus (10). The foot segment is defined by three anatomical markers on the heel (11), the lateral foot (12), and the first metatarsal head (13). During a static calibration trial, additional anatomical markers were added to the medial femoral condyles and the medial malleoli to define the knee and ankle joint axis.

Nine subjects (BMI: 26 ± 5 kg/m^2^, age range: 20–50, both males and females) participated in the study. The experimental protocol was previously described in detail by De Groote et al. (2009). Instrumented gait analysis using a modified Cleveland Clinic marker protocol (34 markers during static trial, 30 markers during gait, see Figure 1) was carried out. The marker trajectories were measured at 200 Hz using a seven-camera motion capture system (Qualysis, Inc., Goteborg, Sweden) during a static trial and during gait at self-selected speed. Ground reaction forces were measured at 2400 Hz by two synchronized force plates (AMTI, Watertown, MA, USA and Bertec, Columbus, OH, USA). Simultaneously, the surface EMG of eight muscles was collected: biceps femoris, rectus femoris, vastus lateralis, semimembranosus, gastrocnemius (medial head), tibialis anterior, soleus, and gluteus medius. The raw EMG signal was band-pass filtered between 10 and 50 Hz using a fourth order Butterworth filter and root mean square values were calculated using a 100 ms time window. A minimum of three valid trials were collected for each limb. From inspection of the measurement data, a representative trial was selected. All procedures were approved by the Stanford University panels on human subjects in research, and all subjects gave informed consent.

### 2.2. Musculoskeletal model

The musculoskeletal model consists of eight segments: a head−arms−trunk (HAT) segment, the pelvis, left and right thigh, lower leg and foot (Delp et al., 1990). The model includes 19 degrees of freedom: spherical joints connect the HAT segment to the pelvis and the pelvis to the thighs. The ankle joints are modeled as simple hinges, whereas the knee joints are modeled as sliding hinges (Yamaguchi and Zajac, 1989). The remaining 6 degrees of freedom correspond to the position and orientation of the pelvis. Each leg is articulated by 43 muscles (see Table 1 for a list of the muscles).

**Table 1:** **Muscles included in the model**.

**Muscle**	
Gmed1/2/3	Gluteus medius anterior/middle/posterior
Gmin1/2/3	Gluteus minimus anterior/middle/posterior
SM	Semimembranosus
ST	Semitendinosus
BFl/s	Biceps femoris long/short head
SAR	Sartorius
ADDl/b	Adductor longus/brevis
ADDm1/2/3	Adductor magnus distal/middle/proximal
TFL	Tensor fasciae latae
PEC	Pectineus
GRA	Gracilis
Gmax1/2/3	Gluteus maximus superior/middle/inferior
IL	Iliacus
PS	Psoas
QF	Quadratus femoris
GEM	Gemelli
PIR	Piriformis
RF	Rectus femoris
VM/I/L	Vastus medialis/intermedius/lateralis
GM/L	Gastrocnemius medialis/lateralis
SOL	Soleus
TP	Tibialis posterior
FLd/h	Flexor digitorum/hallucis longus
TA	Tibialis anterior
PERb/l/t	Peroneus brevis/longus/tertius
EXd/h	Extensor digitorum/hallucis longus

Muscles with a complex geometry such as broad attachments were split in different parts that are controlled independently.

Skeleton dynamics is described by applying the Euler-Lagrange formalism (Craig, 1986):

(1) $$M(q).\overset{..}{q}+c(q,\dot{q})+g(q)-S(q).W_{\text{ext}}=R(q).F_{\text{mt}}.$$

The generalized coordinates **q** describe the motion along the degrees of freedom. **M**(**q**) denotes the generalized inertia matrix, **c**(**q**, $\dot{q}$) the vector of generalized Coriolis and centrifugal forces, and **g**(**q**) the vector of gravitational forces. **W**_ext_ denotes the generalized external forces and **S**(**q**) is the geometric transformation from generalized external forces to generalized joint forces. In the case of gait, the generalized external forces are the ground reaction forces and moments. **F**_mt_ denotes the vector of musculotendon forces and **R**(**q**) is the geometric transformation matrix of the musculotendon forces to joint forces.

Muscle activation and contraction dynamics are neglected. A linear relation between muscle activation and muscle force was assumed:

(2) $$F_{\text{mt}}=a.F_{\text{mt}}^{\text{max}}(q),$$

with *a* muscle activation and *F*^max^_mt_ the instantaneous maximal force generating capacity of the muscle, which was calculated from the muscle's force-length-velocity properties (Zajac, 1989) based on the kinematics.

### 2.3. Calculation of muscle activity

Musculoskeletal models, scaled to the subject's dimensions, were generated using OpenSim (Delp et al., 2007) based on marker information collected during the static trial. Joint kinematics were calculated from the measured marker trajectories during gait using a Kalman smoothing algorithm (De Groote et al., 2008). The Kalman smoothing algorithm for inverse kinematics has been shown to be an accurate method to reconstruct joint kinematics from measured marker positions during gait. The accuracy of this method in the presence of instrumental errors and soft tissue artifacts is higher than the accuracy of traditional methods based on a least-squares fit between measured and modeled marker positions. Subsequently, an inverse dynamic analysis calculated the joint reaction torques **T**_ID_ by evaluating the left hand side of Equation (1):

(3) $$T_{\text{ID}}=M(\tilde{q}).\overset{..}{\tilde{q}}+c(\tilde{q},\dot{\tilde{q}})+g(\tilde{q})-S(\tilde{q}).{\tilde{W}}_{\text{ext}},$$

with $\tilde{q}$ referring to the joint kinematics calculated based on the experimental marker trajectories and $\tilde{W}$_ext_ referring to the experimentally measured ground reaction forces and torques. Joint reaction torques were input to a static optimization algorithm to calculate muscle activations (for details see Anderson and Pandy, 2001; De Groote et al., 2012). At each sample time *t*_*k*_, the static optimization algorithm solves a quadratic optimization problem to calculate muscle activity. The performance criterion is the sum of squared muscle activations:

(4) $$\sum_{m\;=\;1}^{M}a_{m}^{2}(t_{k})$$

with *m* = 1 … *M* referring to the different muscles in the model. This performance criterion is minimized under the constraint that the muscles generate the inverse dynamic joint reaction torques using the linear relation between muscle activation and muscle force expressed by Equation (2):

(5) $$T_{\text{ID}}=R(\tilde{q}).F_{\text{mt}},$$

with **F**_mt_ = [*F*_mt, 1_ … *F*_mt,*M*_]^*T*^ and

(6) $$F_{\text{mt},m}=a_{m}.F_{\text{mt},m}^{\text{max}}(\tilde{q}).$$

Additionally, muscle activations are constrained between 0 and 1. Calculated muscle activations were qualitatively compared to measured EMG.

### 2.4. Synergy analysis

For each subject, the *M* × *K* matrix **A** of calculated activations with *M* the number of muscles and *K* the number of time instants was decomposed in an *M* × *N* matrix **W** and an *N* × *K* matrix **C** with *N* the number of synergies using NNMF (Lee and Sueng, 1999; Ting and Macpherson, 2005). The columns of **W** specify the relative weightings of the muscles in each synergy whereas the rows of **C** specify the activation pattern of each synergy. NNMF is based on an optimization procedure that finds for a pre-specified number of synergies matrices **W** and **C** with non-negative elemants that minimize the sum of squared differences between the actual data (**A**) and the reconstructed muscle activations (**W**.**C**) (cfr. Ting and Macpherson, 2005):

(7) $$\sum_{mk}{\left(A_{mk}-{\left(W.C\right)}_{mk}\right)}^{2}$$

with *mk* referring to the elements in the *m*th row and *k*th column.

We used the algorithm proposed by Lee and Sueng (2001) to solve the optimization problem described above. The algorithm starts from matrices **W**_0_ and **C**_0_ with randomly assigned non-negative initial values. Matrices **W** and **C** are updated in each iteration of the algorithm using multiplicative update rules:

(8) $$C_{i\;+\;1}=C_{i}\frac{W_{i}^{T}A}{W_{i}^{T}W_{i}C_{i}},W_{i\;+\;1}=W_{i}\frac{AC_{i}^{T}}{W_{i}C_{i}C_{i}^{T}},$$

with *i* referring to the iteration. Lee and Sueng (2001) proof that using these update rules **W** and **C** converge to a local optimum. Since the optimization problem underlying NNMF is non-convex, there is no guarantee that the obtained local optimum is also a global optimum. Therefore, the algorithm was repeated for 10 random initial guesses and the solution with the closest correspondence between actual and reconstructed muscle activations was retained.

The NNMF algorithm requires the number of synergies as an input. Since the number of synergies needed to accurately reconstruct the calculated activations was unknown a priori, the NNMF algorithm was repeated for one, two, three, four, five, and six synergies. Determination of the number of synergies needed to reconstruct the calculated activations was based on the variability accounted for (VAF) defined as uncentered Pearson's coefficient of determination. VAF was evaluated globally over all muscles and for each muscle individually. We considered two criteria to determine the number of synergies: (i) a total VAF larger than 90% and (ii) a VAF larger than 75% for at least 40 out of 43 muscles (Ting and Chvatal, 2010).

### 2.5. Comparison of synergies

Both the number of synergies and the composition of the synergies were analyzed. The synergies obtained from the calculated activations were compared to the synergies reported by Clark et al. (2010) based on an NNMF of EMG measured in 20 healthy subjects during walking at self-selected speed. Surface EMG was recorded from eight muscles (tibialis anterior, soleus, medial gastrocnemius, vastus medialis, rectus femoris, medial hamstrings, lateral hamstrings, and gluteus medius) during three seperate trials. EMGs were high-pass filtered (40 Hz), demeaned, rectified, and low-pass filtered (4 Hz). Processed EMG was normalized to its peak value from self-selected walking and resampled at 1% of the gait cycle. The NNMF algorithm was applied for each leg of each subject to an *M*_*EMG*_ × *K*_*EMG*_ matrix with *M*_*EMG*_ = 8, the number of muscles from which EMG was measured, and *K*_*EMG*_ = number of strides × 101, the time base corresponding to all strides from two of the three trials. The number of synergies was determined based on the VAF. The number of synergies was increased until the VAF for all muscles in each of six regions of the gait cycle was above 90% or until adding an additional synergy did not increase VAF by more than 5% for the muscle(s) and/or region(s) with the lowest VAF. Activation patterns were first averaged over the strides within one leg and thereafter over the left and right legs of all subjects. Muscle weightings were averaged over all left and right legs.

Since the analysis of Clark et al. (2010) was based on EMG recordings of a restricted set of eight muscles, only part of the results can be compared. To investigate the effect of the different set of muscles, we repeated the NNMF of the calculated activations with a limited set of muscles corresponding to the muscles included in the analysis of Clark et al. (2010). Muscle weightings of our analysis (with both the full set and the reduced set of muscles) and the analysis of Clark et al. (2010) were compared using Pearson's coefficient of correlation. Coefficients of correlation and the corresponding *p*-values are reported.

### 2.6. Assumptions and limitations

The calculated activations are influenced by measurement and modeling errors. Joint kinematics are assessed indirectly through skin-mounted markers. As a consequence, calculated joint kinematics are influenced by soft tissue artifacts. Although we used an accurate method to estimate joint kinematics from measured marker trajectories, the influence of soft tissue artifacts cannot be completely eliminated. In addition, the musculoskeletal model is a simplified representation of the musculoskeletal system. Most importantly, sensory feedback is not included in the model. Furthermore, model parameters are based on a generic model that is scaled to the subject's dimensions and hence subject-specific features, e.g., musculoskeletal geometry and muscle strength, are not accounted for. In addition, static optimization does not take into acount muscle dynamics and hence the time delays between muscle excitation and force production are not accounted for.

Experimental EMG is subject to measurement noise. Due to the high amount of noise, EMG data is processed. EMG processing generally includes high-pass filtering, rectification, and low-pass filtering. Different cut-off frequencies are being used. It is likely that the filter frequencies influence the EMG-based synergy analysis. For example, the lower low-pass filter frequency in the study of Clark et al. (2010) than in the study of Zelik et al. (2014) (4 Hz vs. 10 Hz) might be one factor explaining the different number of synergies underlying gait found in both studies (on average 3.8 synergies vs. 5.8 synergies).

Selecting a threshold for reconstruction of EMG signals or calculated activations is a difficult and subjective decision. There is no consensus in the literature. Furthermore, Steele et al. (2013) have shown that the number of muscles used in the analysis influences the variability accounted for by a given number of synergies. For example, the lower number of muscles in the study of Clark et al. (2010) than in the study of Zelik et al. (2014) might be another factor explaining the different number of synergies underlying gait found in both studies (on average 3.8 synergies vs. 5.8 synergies), although the higher threshold for the VAF by individual muscles used by Clark et al. (2010) might limit the effect of the number of muscles.

In this study, synergies based on EMG and calculated activations are compared. Part of the differences between both sets of synergies might be due to different levels of noise of the different experimental inputs. In addition, the calculated activations are influenced by modeling errors.

Synergies based on calculated activations and EMG-based synergies are derived from different samples of the same population. Subject-specific differences in gait patterns and muscle control might therefore influence the comparison. To reduce the influence of noise on the EMG, EMG-based synergies are commonly determined based on EMG collected during multiple strides (e.g., Clark et al., 2010; Zelik et al., 2014). Since our experimental data only contained EMG data for single strides, we chose to compare our results to previously reported results.

## 3. Results

**Figure 2:**
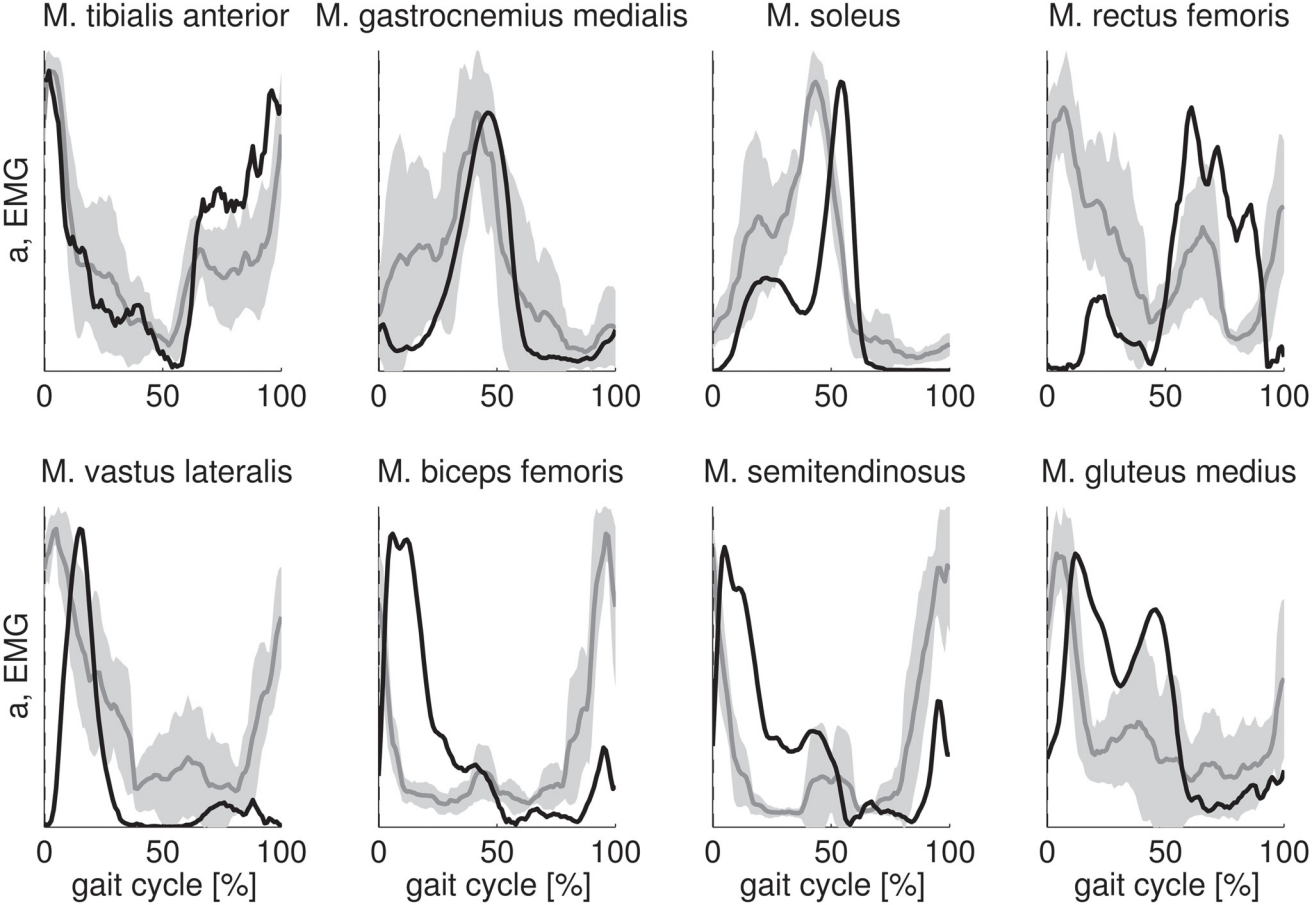
**Comparison of calculated muscle activations (black) with measured EMG (gray)**. Calculated muscle activations and EMG are scaled to the same maximum value. Scaled muscle activations and EMG are expressed as a function of the gait cycle percentage and then averaged over the subjects. The standard deviation of the EMG is indicated by the gray band.

The key features of the EMG of tibialis anterior, gastrocnemius medialis, soleus, and gluteus medius are well-predicted by the calculated activations (Figure 2). The calculated activations for tibialis anterior and gastrocnemius medialis are approximately within one standard deviation of the experimental EMG. Calculated activations for soleus and gluteus medius show a high activation with two peaks during stance similar to EMG but the relative magnitude of the peaks as well as the timing of the peaks are not wel predicted. Activity of vastus lateralis, biceps femoris, and semitendinosus during initial stance is predicted but the calculated activations have a time lag with respect to the EMG. However, the calculations fail to predict the high activity of vastus lateralis, biceps femoris and semitendinosus at the end of swing. For rectus femoris the fit is poor.

On average four (3.7 ± 0.7) synergies were required to reconstruct unilateral lower extremity muscle activations during walking at self-selected speed in accordance with the two criteria described above. Of the nine subjects, four subjects required three synergies, four subjects required four synergies, and one subject required five synergies.

**Figure 3:**
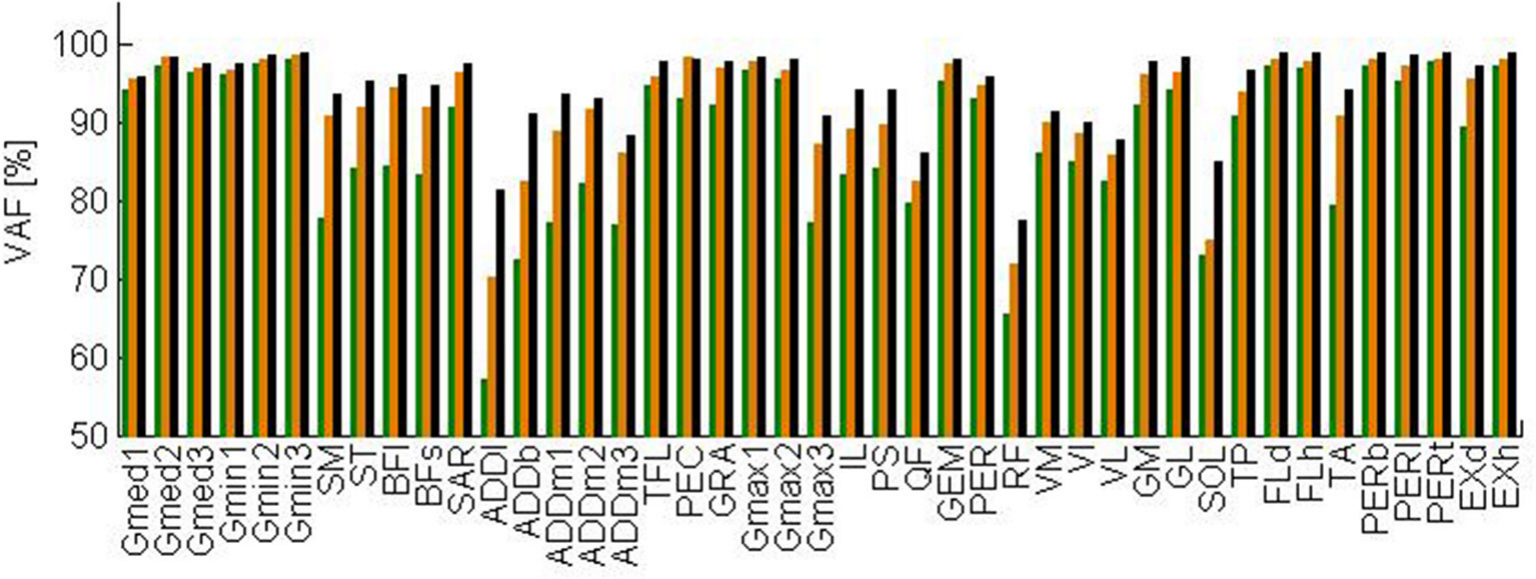
**Variability accounted for by three (green), four (orange), and five (black) synergies extracted by NNMF averaged over the test subjects**. Abbreviations are explained in Table 1.

The total VAF averaged over the test subjects is 91, 94, and 96% when three, four, and five synergies are extracted, respectively. Figure 3 shows the VAF for each muscle averaged over the test subjects for three, four, and five synergies. When extracting four synergies, the average VAF is lower than 75% for rectus femoris, adductor longus, and soleus.

**Figure 4:**
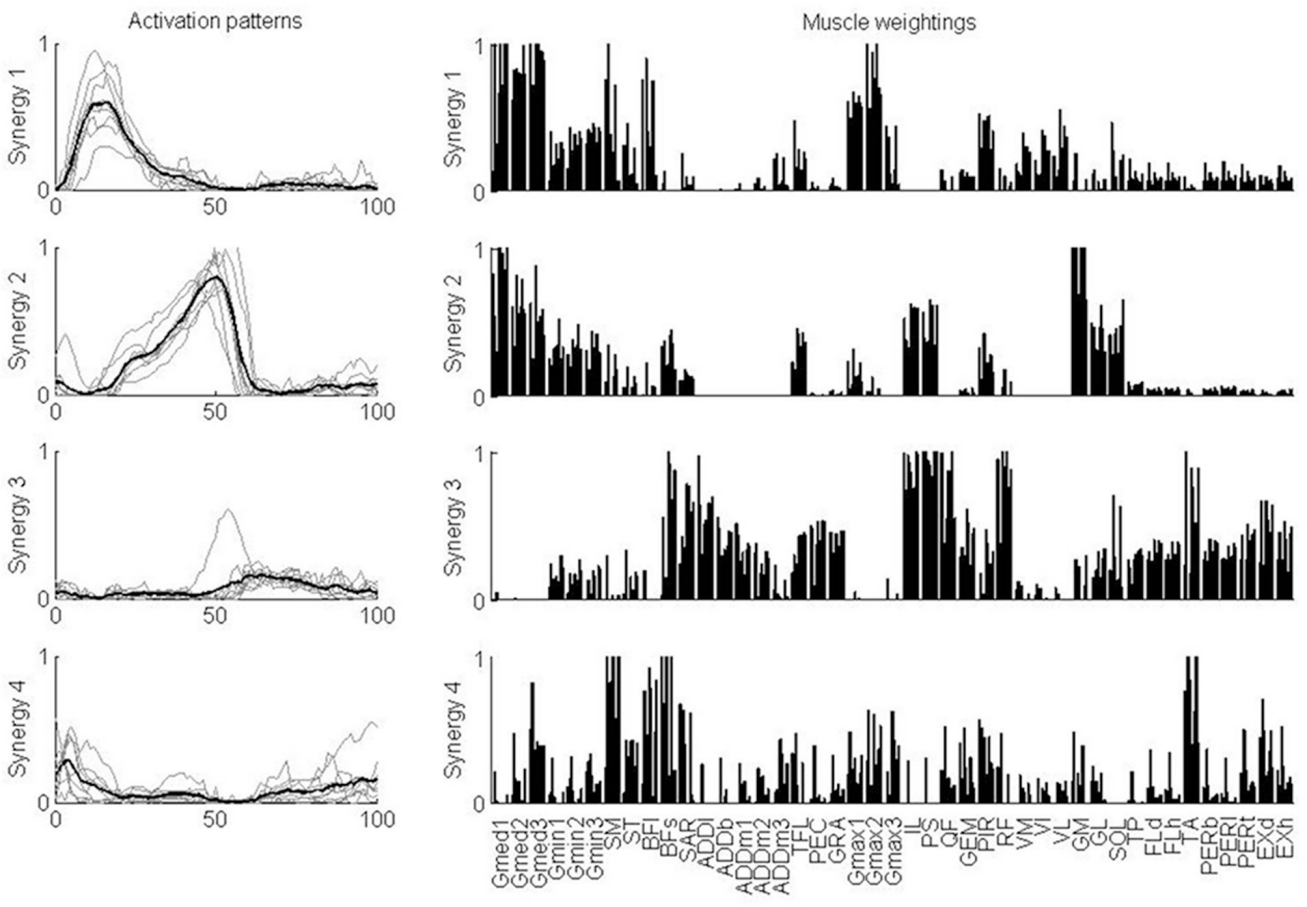
**Synergy activation patterns and muscle weightings for each of the four synergies**. Activation patterns indicate how activation of a synergy varies over the gait cycle. Thin gray lines show the patterns for each individual subject. The thick black line shows the mean over the test subjects. Muscle weightings indicate the relative strength of representation of each muscle in the synergy. For each muscle, the weightings for each of the nine subjects are plotted. Abbreviations are explained in Table 1.

For further analysis, four synergies were considered for each subject, independent of the number of synergies that was determined based on the VAF criteria. An equal number of synergies facilitated the comparison of the activation patterns and muscle weightings across subjects. The characteristics of each synergy were quite similar across the test subjects (Figure 4). Synergy 1 consisted mainly of the glutei, piriformis and vasti. This synergy was activated in early stance. Synergy 2 consisted mainly of gastrocnemii and soleus, although gluteus medius, gluteus minimus, iliacus, and psoas were also represented. This synergy was activated during late stance. Synergy 3 consisted mainly of rectus femoris, tensor fasciae latae, the hip adductors (ADDb, ADDl, ADDm), iliacus, psoas, gracilis, pectineus, flexor, and extensor digitorum and hallucis longus and tibialis posterior although biceps femoris long head and tibialis anterior were also represented. This synergy was activated during (early) swing. Synergy 4 consisted mainly of the hamstrings (ST, SM, BFl, BFs) and tibialis anterior. This synergy was activated during early stance and late swing.

**Figure 5:**
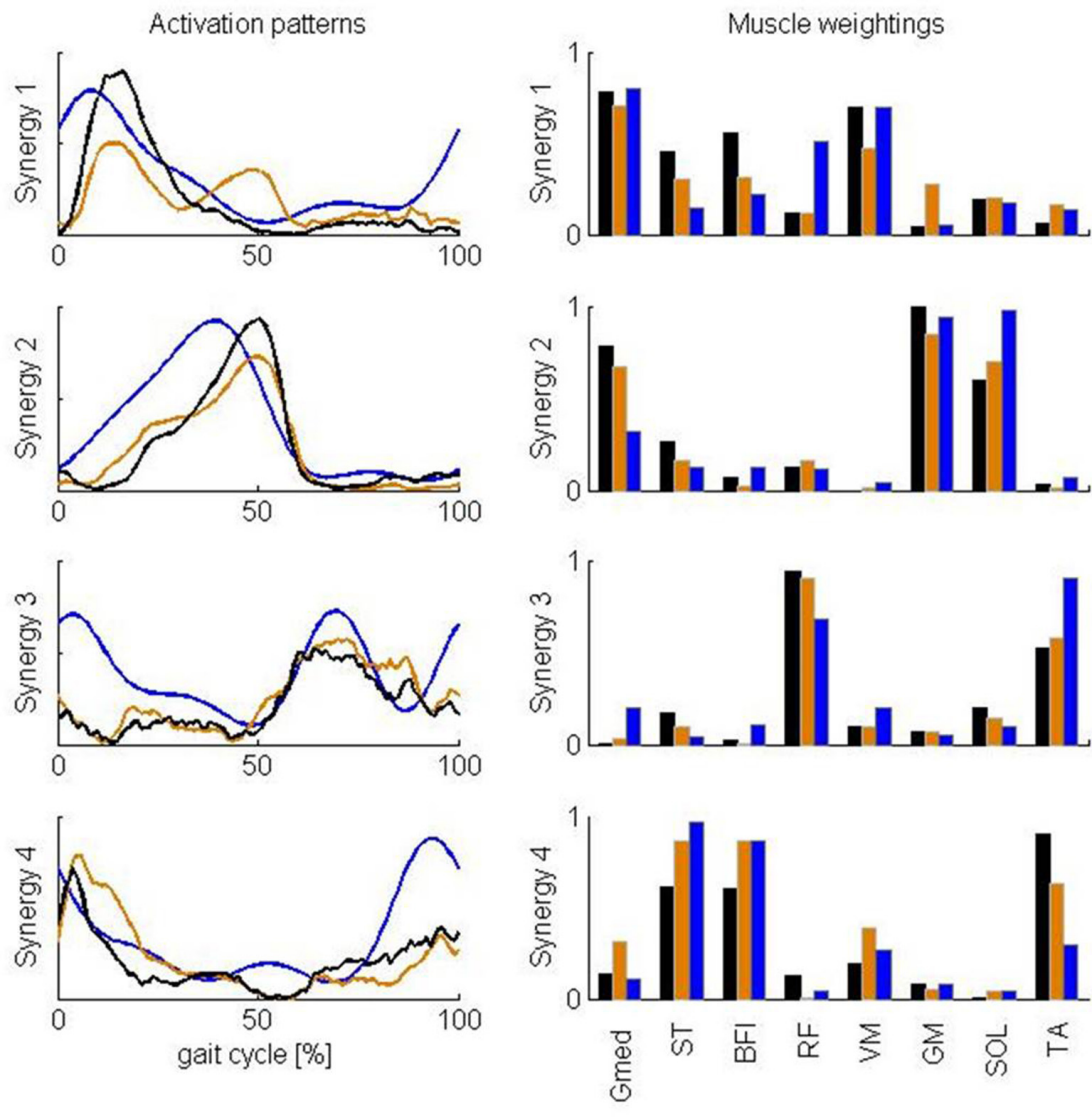
**Comparison of averaged synergy activation patterns and muscle weightings reported by Clark et al. (2010) and calculated in this study based on the full and reduced set of muscles**. The results reported by Clark et al. (2010) for healthy subjects are represented in blue. Results of this study based on all 43 muscles and a subset of the muscles are represented in, respectively, black and orange. Calculated synergies were rescaled to be in better accordance with the results of Clark et al. (2010). After rescaling, the maximum of the reconstructed activity for each of the muscles and for each individual subject is one and the maximum weighting within each synergy is one for the eight muscles considered in this comparison. Abbreviations are explained in Table 1.

Clark et al. (2010) found a similar number of synergies with a similar standard deviation. Activation patterns and muscle weightings for the eight muscles included in the study of Clark et al. (2010) are similar to our results (Figure 5). Two differences are, however, worth noticing. First, the third synergy reported by Clark et al. (2010) is activated both in early stance and early swing whereas in our case this synergy is only activated in early swing. Second, Clark et al. (2010) report a high weight of tibialis anterior in the third synergy and only a small weight in the fourth synergy whereas in our case the weights of tibialis anterior in the third as well as in the fourth synergy are high. We found that Pearson's coefficient of correlation between the muscle weightings of our analysis and the analysis of Clark et al. (2010) increased when the same subset of muscles is used to calculate the synergies (Table 2). Especially, the weight of tibialis anterior in the third synergy increased whereas it decreased in the fourth synergy. The correlation between the muscle weighgings of the analysis of Clark et al. (2010) and our analysis using the same subset of muscles is statistically significant (*p* < 0.05) for three synergies and close to statistically significant for one synergy (*p* = 0.055) whereas statistical significance was found for only two synergies when using all muscles.

**Table 2:** **Pearson's coefficients of correlation *r* and corresponding *p*-values between the muscle weightings reported by Clark et al. (2010) and the muscle weightings we extracted from the calculated muscle activations when, respectively, all and a subset of the muscles were included in our analysis**.

**Synergy**	**All muscles**		**Subset of muscles**	
	***r***	***p***	***r***	***p***
1	0.69	0.061	0.70	0.055
2	0.81	0.013	0.89	0.003
3	0.81	0.016	0.87	0.005
4	0.66	0.072	0.92	0.001

## 4. Discussion

We investigated whether the low-dimensionality of muscle activity that has been observed through the analysis of EMG recorded during walking, can be explained by the combination of task constraints and the minimization of muscle effort. EMG-based analyses of synergies do not allow investigating whether the origin of synergies is neural or task and performance related. In contrast, using a model-based approach we can combine independent recruitment of the muscles with task constraints and performance optimization, and hence study the effect of task constraints and performance optimization in the absence of synergistic muscle control. We therefore calculated muscle activations producing measured gait kinematics while minimizing muscle effort based on a musculoskeletal model with 43 muscles per leg that could be recruited independently. Using NNMF we found that the dimensionality of the calculated muscle activations was low. Three to five modules accounted for over 90% of the total variability. This is in accordance with the numbers of synergies previously reported for walking (Patla, 1985; Davis and Vaughan, 1993; Olree and Vaughan, 1995; Ivanenko et al., 2004; Clark et al., 2010). Hence we can conclude that task constraints (walking kinematics and reaction forces between the ground and the feet were input to the calculations) in combination with minimization of muscle effort can explain the low-dimensionality of muscle activity.

The numbers of synergies we extracted from the calculated excitations are very similar to the corresponding results reported by Clark et al. (2010). The value of the VAF by the individual muscles used to determine the number of synergies was lower in this study than in the study of Clark et al. (2010). Steele et al. (2013), however, demonstrated that experimental analyses that include fewer muscles may over-estimate the variance accounted for compared to an analysis that included all the muscles involved in the task. We therefore think that the difference in number of muscles in the analysis (43 in our analysis vs. eight in the analysis of Clark et al.) motivates the choice of a lower value for the VAF in our case.

Activation patterns and muscle weightings we extracted from the calculated activations are very similar to the corresponding results reported by Clark et al. (2010) except for two main differences. First, the third synergy reported by Clark et al. (2010) is activated both in early stance and early swing whereas in our case this synergy is only activated in early swing. Second, Clark et al. (2010) report a high weight of tibialis anterior in the third synergy and only a small weight in the fourth synergy whereas in our case the weights of tibialis anterior in the third as well as in the fourth synergy are high. Differences might arise from modeling errors and the different number of muscles in both analyses. First, the model and performance criterion used to calculate muscle activations are only an approximation of the true musculoskeletal system. The modeling errors are reflected in the differences between calculated activations and EMG. We fail to predict the high activity of rectus femoris during early stance. A possible reason for the difficulty of calculating rectus femoris activity is that this muscle presumably has activity which depends heavily on afferent input and reflexes, which were not included in the model. For example, in cats the activity in RF differed between fictive locomotion (i.e., in absence of reflexes) and normal forward level walking, indicating that afferent input helps shaping the activity profile of this muscle during locomotor activity (Markin et al., 2012). Given this mismatch between calculated and measured RF activity, it is not surprising that we do not find activity during early stance in the third synergy which is dominated by rectus femoris. Similarly hamstring activation during terminal swing depends on afferent input and the lack of reflex activation in our model can explain the lower activation in the fourth synergy dominated by the hamstrings during terminal swing. Further, the calculated activations have a time lag with respect to the EMG due to neglecting muscle activation and contraction dynamics in our model. This time lag of calculated activations with respect to EMG is also reflected in the activation patterns where the peak in activation in the first, second, and fourth pattern extracted from the calculated activations is delayed with respect to the corresponding peak in the EMG-based patterns. Second, Steele et al. (2013) showed that the number of muscles used to calculate synergies influences the muscle weightings and activation patterns within the synergies. When we repeated the NNMF with a limited set of muscles corresponding to the muscles included in the analysis of Clark et al. (2010), we found that Pearson's coefficient of correlation between the muscle weightings of our analysis and the analysis of Clark et al. (2010) increased. Especially, the weight of tibialis anterior in the third synergy increased whereas it decreased in the fourth synergy. Based on the observed similarities, even in the presence of the confounding factors discussed above, we conclude that task constraints and the optimization of muscle effort not only explain the low-dimensionality of the muscle activity but also the activation patterns and muscle weightings within the synergies determined from EMG recordings.

Although Davis and Vaughan (1993) and Ivanenko et al. (2004) used a different decomposition technique (factor analysis) to extract muscle synergies from EMG, they reported a similar composition and similar activation patterns than Clark et al. (2010). Hence, a comparison based on their results would have led to the same conclusion but would have been a little less straightforward since factor analysis also allows negative weights.

The reported similarity between muscle synergies obtained from decomposing calculated activations at the one hand and measured EMG at the other hand during gait is in accordance with Steele et al. (2013) who investigated the muscle activity underlying an isometric force task at the hand. They found that the muscle synergies calculated from the musculoskeletal model were similar to the experimental synergies whereas the model assumed independent control of muscles. They, however, used a predefined number of four synergies and found a lower total VAF when all 29 muscles in their model were included in the analysis.

Our results might seem to contradict the results of Borzelli et al. (2013) who found that calculated muscle activations are in better accordance with experimental EMG for minimum effort recruitment of synergies than for minimum effort recruitment of individual muscles for an isometric force task at the hand. Based on this result, Borzelli et al. (2013) suggest that the central nervous system generates forces at the hand by recruitment of muscle synergies rather than by recruitment of individual muscles. Their synergy-based calculation, however, is based on synergies extracted from measured EMG and uses the EMG-based muscle weightings as an input while calculating the activation patterns. Hence, the synergy-based calculation uses information from the EMG recordings while the calculation based on independent control of individual muscles does not use a priori information. Therefore, differences between both methods cannot be solely attributed to differences in the muscle control strategy but need to be attributed to differences in the experimental input data as well. For this reason, the comparison of activations calculated using both methods with the EMG is biased toward the synergy-based calculation. Nevertheless, the results of Borzelli et al. (2013) show that synergistic muscle control and performance optimization are compatible. As a consequence, it is possible to use a synergy-based description of muscle activity based on the measured EMG to augment the reliability of the calculations and to reduce the influence of modeling errors. This is in accordance with a previous study of balancing in the cat (McKay and Ting, 2012) and was recently also confirmed for gait (Walter et al., 2014). Our finding that task constraints (joint kinematics and external forces) and performance optimization result in low-dimensional synergistic muscle action is in accordance with the observation that experimental EMG collected for a limited set of superficial muscles can be successfully used to constrain the activations of all muscles.

The present data have implications for current concepts of the neural control of gait. Indeed, the results make it increasingly clear that one should see synergies as task-defined entities (Ivanenko et al., 2013). Hence the primary benefit is that one can start to understand gait in terms of a set of sub-tasks, the combination of which leads to an optimum solution which we call gait. In this context it should be emphasized that the synergies, supporting these sub-tasks, are the result of a combination of inputs, from spinal cord central pattern generators, afferent input and supraspinal sources (Ivanenko et al., 2013). It follows that it may be erroneous to expect to find cells or circuits for each of the defined synergies entities (in the same sense that it is useless to look for “grandmother cells” in the cortex). In neural terms there is only evidence for one or two basic neural synergies (i.e., the flexor reflex synergy; see Duysens et al., 2013) related to gait. Not surprisingly, these basic synergies show up only in the very early phases of development of gait (Dominici et al., 2011), or in studies of reduced cat preparations, when the basic locomotor pattern returns to a simple alternation of activity in flexors and extensors (Duysens, 1977). Similarly, in patients with spinal cord or brain lesion there is a reduction of basic components during walking (Ivanenko et al., 2003; Clark et al., 2010). Admittedly, the present data do not falsify the hypothesis that in intact humans the central nervous system produces walking through the activation of a more extensive set of four or five neural synergies. However, we demonstrated that there is no need to think in these terms, in particular because of the large plasticity and flexibility of the central nervous system. Given the measured kinematics, no reference to fixed neural synergies is needed to reconstruct the synergies observed through analysis of EMG.

D'Avella and Pai (2010) argue that unlike a non-modular controller, a modular controller must adapt faster to a perturbation that is compatible with the modules than to an incompatible perturbation. Berger et al. (2013) indeed find that adaptation to a compatible perturbation is faster. Our study confirms modular muscle action and suggests that the modularity might be driven by performance optimization. It would therefore be interesting to investigate whether the modules that evolve from an incompatible perturbation optimize performance.

Although the methodological limitations discussed in Section 2.6 might influence our quantitative results, it is unlikely that they would influence the conclusion that synergies derived from calculated activations are similar to EMG-based synergies. In this study, we compared two sets of synergies derived from experimental inputs with different levels of measurement noise. Noise introduces differences and therefore reduction of noise is expected to increase the similarities and hence to strengthen the conclusion. In addition, the synergies compared in this study are based on different samples from the same population (walking at self-selected speed in healthy adults). Again, the use of different samples introduces differences and therefore use of the same samples is expected to increase the similarities and hence to strengthen the conclusion. The evaluation criteria used to determine the number of synergies are subjective. Our choices have an influence on the number of synergies. Therefore, care should be taken when comparing the number of synergies between studies. For this reason, we did not aim at determining the significance of the similarity of the number of synergies in our study and the study of Clark et al. (2010). Since for the comparison of activation patterns and muscle weightings a fixed number of synergies was used, the evaluation criteria do not influence that part of the study.

Since our analysis is based on experimental kinematics and external forces, we could only assess the dimensionality of muscle recruitment underlying actual human walking. The studied walking patterns are the result of a locomotor strategy. Studying the dimensionality of the locomotor strategy, in contrast to the dimensionality of muscle recruitment underlying an actual gait pattern, would require a more general (high level) definition of task constraints, e.g., displacing the center of mass at a given speed while maintaining stability. An analysis based on a high level definition of task constraints would require different simulation techniques than the inverse dynamic approach used in this study. Simulation techniques allowing to study motion synthesis, however, are not well established.

In conclusion, we have demonstrated that task constraints and the minimization of muscle effort explain the number of EMG-based synergies as well as the composition of EMG-based synergies underlying a given walking pattern in healthy subjects. Our mapping from experimentally measured gait motion to muscle activations that was based on independent control of individual muscles, revealed muscle synergies similar to those that are observed through analysis of EMG. Hence, our results suggest that modeling synergistic muscle control would not further reduce the dimensionality of muscle activities calculated using an inverse approach combined with effort minimization. Our analysis, however, does not allow falsifying the hypothesis that the central nervous system produces walking through the activation of a small number of muscle synergies. First, the similarity in synergy dimensionality and structure might simply indicate that the central nervous system organizes synergies so that the resulting muscle activations during walking are close to those that would be obtained by effort minimization of individually recruited muscles. Second, due to measurement noise and the different data processing underlying both approaches we are unable to rule out that there are small but significant differences. But we illustrated that the EMG-based approach to study muscle synergies cannot be used to support the hypothesis that muscle synergies reflect a motor control strategy.

### Conflict of interest statement

The authors declare that the research was conducted in the absence of any commercial or financial relationships that could be construed as a potential conflict of interest.
